# Rational Design of a Multipurpose Bioadhesive Vaginal Film for Co-Delivery of Dapivirine and Levonorgestrel

**DOI:** 10.3390/pharmaceutics12010001

**Published:** 2019-12-18

**Authors:** Jing Li, Galit Regev, Sravan Kumar Patel, Dorothy Patton, Yvonne Sweeney, Philip Graebing, Sheila Grab, Lin Wang, Vinayak Sant, Lisa C. Rohan

**Affiliations:** 1 Department of Pharmaceutical Sciences, School of Pharmacy, University of Pittsburgh, Pittsburgh, PA 15213, USA; jil132@pitt.edu (J.L.); gar31@pitt.edu (G.R.); patels10@mwri.magee.edu (S.K.P.); smgrab@pitt.edu (S.G.); VIS45@pitt.edu (V.S.); 2 Magee-Womens Research Institute, Pittsburgh, PA 15213, USA; pgraebing@mwri.magee.edu (P.G.); lwang@mwri.magee.edu (L.W.); 3 Department of Obstetrics and Gynecology, University of Washington, Seattle, WA 98195, USA; dpatton@uw.edu (D.P.); ytcs@uw.edu (Y.S.)

**Keywords:** multipurpose prevention technologies, dapivirine, levonorgestrel, bioadhesive vaginal film, HIV, unintended pregnancy, contraception

## Abstract

Human immunodeficiency virus (HIV) infection and unintended pregnancy, which can lead to life-threatening complications, are two major burdens for female reproductive health. To address these pressing health issues, multipurpose prevention technologies (MPTs) are proposed to deliver two or more drugs simultaneously. MPTs could offer several benefits for users such as improved convenience, increased effectiveness, reduced cost, and decreased environmental burden. Here, we report the development, and in vitro and in vivo assessment of a bioadhesive vaginal film as a coitally-independent MPT dosage form for delivering dapivirine (DPV) and levonorgestrel (LNG) to prevent HIV infection and unintended pregnancy, respectively. After confirming the feasibility of bioadhesive film use for weekly drug delivery in vivo through colpophotography and MRI evaluation, the pharmacokinetics (PK) of DPV/LNG single entity and combination bioadhesive films was investigated in pigtailed macaques (*n* = 5). Both drugs from single entity or combination films were able to provide sustained drug release in vivo. The combination film showed lower local tissue clearance for DPV and exhibited significantly increased plasma concentration for LNG as compared to the single entity film. This proof-of-concept study demonstrates the ability of this novel bioadhesive film platform to deliver LNG and DPV simultaneously as an MPT product for the prevention of HIV infection and unintended pregnancy.

## 1. Introduction

In 2010 to 2014, approximately 44% of global pregnancies were unintended or mistimed [1]. This percentage is even higher in some developing countries such as South Africa [2], where unfortunately a high incidence of human immunodeficiency virus infection type-1 (HIV-1) infection is also noted, mostly due to unprotected sexual intercourse [3]. Given that more than 90% of infections among children are transmitted from their HIV positive mother [4], consequential severe health burden may be encountered when women at high risk of HIV infection become pregnant. In the duet microbicide trials conducted in Southern Africa, participants including women, men, health professionals, and community stakeholders indicated strong interest in a product that combined prevention of HIV and prevention of pregnancy [5]. Therefore, it is believed that the design of products that combine HIV prevention agents with contraceptives may lead to enhanced product uptake and effectiveness.

Current existing multipurpose prevention technology (MPT) barrier methods include male and female condoms, diaphragms, and cervical caps. These MPT products provide protection for unintended pregnancy and sexually transmitted infections (STIs) including HIV-1 if they are used correctly and consistently [6,7,8,9,10]. However, in practical use, the condom failure rate is much higher than expected, largely due to women’s inability to negotiate their use with their male partners in many cases [11]. Therefore, women need a new generation of safe and self-initiated MPT products to protect themselves from unintended pregnancy and HIV infection.

A large number of MPTs are currently under development, including intravaginal rings for the delivery of anti-retroviral drugs and contraceptives. A three-month vaginal ring containing dapivirine (DPV) and levonorgestrel (LNG) is under clinical evaluation (MTN-044) for safety and pharmacokinetics (PK) [12]. The DPV-only vaginal ring has shown effectiveness in HIV prevention in phase III trials, and has exhibited potentially higher levels of protection with higher adherence [13]. However, a few women who used vaginal rings reported some issues such as ring expulsion [14,15] and detection by their male partners [16,17]. Ultimately, availability of multiple product options for MPTs would provide women with choices and may increase uptake by women.

The polymeric film as a vaginal dosage form has great potential for development as an MPT product platform. Films have low manufacturing cost, making them affordable for use in women in low-income settings and developing countries. The film is also a portable and discreet dosage form. Their portability is an important consideration for global distribution. Due to these advantages, multiple anti-retroviral drugs including DPV have been successfully formulated into polymeric films as topical microbicides for HIV prevention [18,19,20,21,22]. Their user acceptability, safety, pharmacokinetics, (PK) and pharmacodynamics (PD) (ex vivo challenge) have been evaluated in clinical studies [21,23,24]. It was observed that the film platform showed high acceptability, excellent safety profile for vaginal administration, and favorable PK and PD properties. However, to the best of our knowledge, exploration of the polymeric film as an advanced functional vaginal drug delivery platform featuring mucoadhesiveness and controlled release has not yet been explored.

Thiolated polymers were reported to bind to mucin strongly due to covalent bond formation [25]. Excellent tolerance and safety of thiomer-containing dosage forms has been demonstrated in animals [26,27] and clinical trials [28]. In addition, the features of thiomers such as in situ gelling [29], controlled release [30,31], tissue permeation enhancement [32], and maximal mucoadhesion around vaginal pH of 3.8–4.5 [33] makes them highly advantageous for intravaginal drug delivery. In our study, a clinically evaluated polyvinyl alcohol (PVA)-based film was modified through addition of thiomers to improve its tissue residence and extend drug release. These features were incorporated by design to develop a vaginal film product with decreased dosing frequency.

DPV and LNG were selected as the model drugs for designing the MPT film platform. DPV, a non-nucleoside reverse transcriptase inhibitor, has been evaluated in multiple clinical trials in different dosage forms, such as gels, polymeric films, and vaginal rings, for prevention of sexually transmitted HIV [24,34]. Hormonal contraceptives have been utilized to decrease the risk of unintended pregnancy since 1969. LNG is a progestin contraceptive drug which is commercially available in oral and intrauterine device formats. Its efficacy and safety has been previously demonstrated [35]. For this reason, LNG has been evaluated as the hormonal contraceptive component in several MPT products under investigation. When developing an MPT product, it is critical to investigate potential drug–drug interactions and their impact on PK and PD properties [36]. No diminished effectiveness of contraception was found when oral LNG was used with the DPV vaginal ring in the clinic [37]. However, the co-delivery of these drugs in a vaginal film has not been evaluated with respect to their local PK. Therefore, it is worthwhile to investigate these properties for this combination in the vaginal polymeric film.

The aim of this proof-of-concept study was to describe the development of an innovative bioadhesive film platform for combined delivery of an antiretroviral drug, DPV, and a progestin, LNG. The MPT film was evaluated in pigtailed macaques, demonstrating that both agents can be delivered in a sustained fashion to provide adequate local (cervicovaginal lavages and tissue) and systemic drug concentrations required for therapeutic efficacy.

## 2. Materials and Methods

### 2.1. Materials

DPV was provided by the International Partnership for Microbicides (IPM, Silver Spring, MD, USA). LNG was purchased from CHEMO (Chatam, NJ, USA). Polyethylene glycol 8000 (PEG 8000) was purchased from Spectrum (Gardena, CA, USA). Hydroxypropyl methyl cellulose E5 (METHOCEL E5) was obtained from Dow Chemical Company (Midland, MI, USA). Polyvinyl alcohol 4-88 was purchased from Millipore Sigma (Temecula, CA, USA). Ultrapure water was prepared by passing distilled water through a Milli-Q Reagent Water System (Millipore). Blue dye, gadobenate dimeglumine, and 2-immunothiolane was purchased from Fisher Scientific (Pittsburgh, PA, USA). Porcine gastric mucin and sodium starch glycolate were purchased from Sigma-Aldrich (Carlsbad, CA, USA). All the other chemical reagents for film preparation, as well as mobile phase solvents, were purchased from Fisher Scientific (Pittsburgh, PA, USA).

### 2.2. Methods

#### 2.2.1. Synthesis and Characterization of Thiomer

Thiomer was synthesized following the method reported by Dr. Andreas Bernkop-Schnürch’s group with minor modification [38]. A total of 1 g of chitosan (medium molecular weight around 300 kDa) was dissolved in 100 mL of 1% (*v*/*v*) acetic acid. Premeasured 2-iminothiolane HCl was added to the chitosan solution and pH was adjusted to 5, 6, or 7 with 5 M NaOH. The reaction mixture was continuously stirred for 24 h. The solution was dialyzed multiple times against 5 mM HCl, 5 mM HCl containing 1% NaCl, and 1 mM HCl using dialysis tubing with 10 K molecular weight-cutoff (MWCO). The final solution was lyophilized and stored at 4 °C for future use.

The degree of thiolation on chitosan was determined by quantifying the amount of thiol groups on the modified chitosan with Ellman’s reagent. A total of 5 mg of thiomer was dissolved in 2.5 mL Milli-Q water. The Ellman’s reagent was prepared by dissolving 3 mg of 5,5′-dithiobis (2-nitrobenzoic acid) in 10 mL of 0.5 M phosphate buffer, pH 8.0. Then, 250 µL of 0.5 M phosphate buffer, pH 8.0, and 500 µL of Ellman’s reagent were added to the above thiomer solution and incubated for 2 h. Afterwards, samples were centrifuged at 12,000× *g* for 5 min to remove precipitated polymers. A total of 250 µL of the reacted supernatant was transferred to a microtitration plate to measure absorbance immediately at a wavelength of 450 nm. The standard curve was prepared by dissolving L-cysteine HCl in chitosan solution by serial dilution (1–1000 µM). The absorbance was tested by the same method as described above. The amount of thiol groups in the thiolated chitosan was calculated on the basis of the standard curve.

#### 2.2.2. Mucin and Polymer Interaction

Thiomers (12% (*w*/*v*)) were hydrated in Milli-Q water and diluted with equal volume of 0.1 M phosphate buffer (pH 6.8) to obtain a concentration of thiomer of 6% (*w*/*v*). The prepared thiomer solution was mixed with equal volume of 8% (*w*/*v*) porcine gastric mucin and stirred gently using a spatula [39]. The solution pH was adjusted to 7 using 2 M NaOH. This solution was incubated for 20 min at room temperature. After incubation, 1% (*w*/*v*) of solid cysteine was added with stirring, and the pH was adjusted to 7. This solution was incubated for 20 min at room temperature. Then, the polymer-mucin incubates were transferred to a viscometer and equilibrated on the plate for 3 min at 25 ± 0.5 °C. Rheological profile was determined using a cone/plate 52 (CP52) spindle on a cone/plate Brookfield model HADVIII+ viscometer (Brookfield Eng. Lab., Inc., Middleboro, MA, USA). Data were collected using Rheocalc software (Brookfield Eng. Lab., Inc. Middleboro, MA, USA). Plastic viscosity and yield stress were calculated by Rheocalc Software using the Casson equation, which gave the best fit. Three controls, thiomer solution without cysteine and chitosan solution with/without cysteine, were prepared using the same method.

#### 2.2.3. Tissue Toxicity of Thiomer

Human ectocervical tissue was obtained from Magee-Womens Hospital Tissue Procurement Center (University of Pittsburgh Medical Center, Pittsburgh, PA, USA) under the protocols approved by the Institutional Review Board (PRO09110431, approved: 8 December 2009). All tissues used in this work were collected from premenopausal women undergoing hysterectomy. The excised tissues were transferred to the lab in Dulbecco’s modified Eagle’s medium (DMEM) and then snap frozen using dry ice and methanol, and stored at −80 °C for later use.

The tissue toxicity of thiomer was evaluated using 3-(4,5-dimethylthiazol-2-yl)-2,5-diphenyl tetrazolium bromide (MTT) assay. The frozen tissue was thawed in a 37 °C water bath for 5 min. The stromal layer was removed from the excised ectocervical tissue using a Thomas Stadie-Riggs tissue slicer (Thomas Scientific, Swedesboro, NJ, USA). Thickness of the tissue was measured using a digital micrometer, and 6 mm biopsies of the ectocervical tissue were obtained using biopsy punch (Integra York PA Inc., York, PA, USA). Biopsies were incubated with 1 mL of DMEM (negative control), 1% *w*/*v* thiomers in DMEM, or 0.6% *w*/*v* of formulated nonoxynol-9 (N-9, GYNOL II; positive control), and shaken in a 37 °C water bath for 6 h. After 6 h, 200 µL of 0.5 mg/mL MTT solution was added to the treated tissues and incubated in a 37 °C water bath for 3 h. The MTT solution in the tissue samples was then replaced with 1 mL methanol and incubated overnight in the dark at room temperature. Tissues were removed from the methanol and dried using a paper towel prior to measuring the tissue weight. The methanol solutions were collected for absorbance measurement at 595 nm. The percentage of viability of tissues was presented as (abs-blank)/mg of tissue as compared to that of DMEM control.

#### 2.2.4. High-Performance Liquid Chromatography (HPLC) for Simultaneous Detection of DPV and LNG

A Waters HPLC system with a 600 series pump, 2487 ultraviolet (UV) detector, and an Empower data acquisition system was used for quantitative analysis. The Waters Sunfire C8 HPLC column (2.5 µm, 4.6 × 50 mm) was used to separate DPV and LNG. The mobile phase consisted of (A) 0.1% of trifluoroacetic acid in Milli-Q water and (B) 0.1% of trifluoroacetic acid in acetonitrile. The method used a gradient elution of 10% B initially, 10% B to 70% B from 0.7 to 4 min with linear change, and then back to 10% B at 7 min with linear change, remaining at 10% B between 7 and 30 min. The flow rate was 2 mL/min. Sample injection volume was 10 µL and both drugs were measured using a wavelength of 254 nm. The autosampler was kept at 4 °C.

#### 2.2.5. Liquid Chromatography-Mass Spectrometry/Mass Spectrometry (LC-MS/MS) for the Detection of Bioanalytical Samples

Quantitation of DPV and LNG in macaque samples was conducted using LC-MS/MS. Specifically, a Thermo UltiMate3000 ultra performance liquid chromatography (UPLC) system coupled with a Thermo TSQ Quantum Access MAX mass spectrometer (Thermo Finnigan, San Jose, CA, USA) was used. The LC separation was carried out on a Phenomenex Hyperclone base deactivated silica (BDS) C8 (3 µm, 4.6 × 150 mm) column. This method was developed for quantification of DPV and LNG in swab, vaginal, and cervical tissues, as well as plasma samples. The mobile phase consisted of (A) 5 mM ammonium formate in 60% acetonitrile and (B) 5 mM ammonium formate in 80% acetonitrile, using a gradient elution with mobile phase B starting from 0% of to 100% over 1.5 min and held at 100% for 2 min and then equilibrated back to 0% for the remaining 2.5 min. All samples contained an internal standard, deuterated dapivirine (d4-DPV). The flow rate was 1.0 mL/min and injection volume was 40 µL. The column was maintained at 40 °C. The column outlet was directly coupled to the heated electrospray ionization sample inlet of the mass spectrometer using positive selective reaction monitoring scan. A spray voltage of 3000 V was applied to the electrospray ionization (ESI) needle. The mass transitions were 330.2/158.0 for DPV, 334/145 for d4-DPV, and 313.1/245.1 for LNG, using collision energies of 25 V. The scan width (*m*/*z*) was set at 0.6, and the scan time was set at 0.5 s. An XCalibur software package (Thermo Fisher Scientific, Waltham, MA, USA) was used for acquiring data.

#### 2.2.6. Film Formulation Development and Physicochemical Characterization

Bioadhesive film was manufactured using a solvent-casting method as previously described with minor modification [21]. Table 1 lists the composition of films. Briefly, thiomer was wetted in Milli-Q water first and stirred until uniform. Polyvinyl alcohol 40-88, polyethylene glycol 8000, and Methocel E5 were dissolved in Milli-Q water and then mixed with the thiomer solution until uniform. DPV and/or LNG were initially dispersed in glycerin and propylene glycol, and then mixed with the polymer solution. The solution was casted onto a polyester substrate using an automatic film applicator (Elcometer 4340) with a 4″ doctor blade. The film sheet was allowed to dry for 15 min at 72 °C before removal from the substrate. Once film sheets were obtained, they were cut using a die press into 1″ × 1″ individual unit doses.

A quick-dissolving film was made as a control using the same manufacturing method. The film formulation is shown in Table 1. This film consisted of the same excipients except that the thiolated chitosan was replaced with the superdisintegrant sodium starch glycolate to impart quick-dissolving properties. Sodium starch glycolate is a common superdisintegrant used in oral dosage forms such as tablets and polymeric films. The majority of vaginal films, especially those intended for vaginal drug delivery of agents against STIs, incorporate faster disintegration and rapid release functionality in order to provide quicker onset of action. Therefore, a quick-dissolving film prepared using sodium starch glycolate served as a comparator to assess thiomer’s in vitro release and in vivo retention.

For both bioadhesive and quick-dissolving films, to aid in visualization of in vivo film retention by colposcopy, a water-soluble blue colored dye (Federal Food, Drug, and Cosmetic Act (FD&C)) was incorporated into the films. Further, a magnetic resonance imaging (MRI) contrast agent (gadobenate dimeglumine) was incorporated to assess film distribution using in vivo contrast-enhanced MRI in macaques. To prepare these films, the dye or gadobenate dimeglumine were first dissolved in the aqueous phase and then mixed with the polymer solution. Film manufacturing remained the same as described above.

To quantify drug content, DPV/LNG films were dissolved in 50% acetonitrile and heated to 60 °C to ensure that the film matrix was completely dissolved. An aliquot was withdrawn and centrifuged at 12,000 rpm for 10 min. The supernatant was further diluted and analyzed by HPLC as described above. The water content, puncture strength, and disintegration of all films manufactured in this study were tested using published methods or methods developed by our group, as reported previously [18,20,21,40,41].

#### 2.2.7. In Vitro Dissolution Test

To compare the drug dissolution between DPV containing bioadhesive and quick-dissolving films, a United States Pharmacopeia (USP) IV apparatus (SOTAX CP7, Horsham, PA, USA) was used. Sink condition for DPV was maintained using 60 mL of 1% (*w*/*v*) cremophor in Milli-Q water as the dissolution medium. The drug release was monitored at 37 °C for 4 h to evaluate drug release kinetics. The amount of drug released was determined by online analysis of the samples at predetermined time intervals using an in-line UV-VIS system (Evolution 300, Thermo fisher, Waltham, MA, USA) at 254 nm.

To assess drug release from LNG-containing single entity and combination (DPV/LNG) bioadhesive films, a USP IV apparatus was used. LNG has poor solubility in 1% cremophor solution. Therefore, to maintain the sink conditions for both DPV and LNG, 40% acetonitrile solution was utilized as the dissolution medium. DPV-containing single entity and combination films were also assessed using this method in order to obtain release comparisons using the same dissolution media as that used for LNG. The drug release was monitored at 37 °C for 60 min. Then, 0.5 mL samples were collected at predetermined time intervals and analyzed by HPLC as described above to quantify the amount of released DPV or LNG.

#### 2.2.8. Ex Vivo Tissue Mucoadhesion

The ex vivo mucoadhesion testing of films was tested using a texture analyzer (TA. XT. Plus, New York, NY, USA) with a 5 kg load cell. The film was attached to the head of a cylinder probe (1″ radius, TA-57R) by double-sided adhesive tape. Porcine intestinal tissue was fixed in the tissue holder with an exposure area of 0.785 cm^2^. Before the probe with the film contacted the tissue, 15 µL of vaginal fluid simulant (VFS) [42] was added to the surface of the tissue and allowed to equilibrate for 15 s. Following equilibration, the probe with the film was moved toward the tissue while constantly applying 150 g force for 60 secs. The probe was withdrawn to detach from the tissue at a speed of 0.5 mm/s. The change of the withdrawal force involved in detaching the film from tissue and the debonding distance were recorded by the Exponent software. The work of adhesion calculated from the area under the force versus distance curve was used to compare the mucoadhesiveness of different film formulations.

#### 2.2.9. In Vivo Evaluations in Macaques

##### Film Retention and In Vivo Drug Release

Five sexually mature female pigtailed macaques (*Macaca nemestrina*) weighing 6–10 kg were used in this study. All five animals were of breeding age and housed at the Washington National Primate Research Center (WaNPRC). All procedures pertaining to the use of these animals were approved by the Institutional Animal Care and Use Committee of University of Washington (IACUC Protocol 2195-18, approved: 20 May 2013), and experiments were performed in accordance with the National Institutes of Health’s laboratory animal use guidelines. Two types of 1″ × 1″ blue dye-containing films were evaluated in this study: DPV (1.41 mg/film) bioadhesive films and DPV (1.51 mg/film) quick-dissolving films. Films were intravaginally inserted at time 0. Colpophotography was utilized to visualize the presence of the film before and after film insertion for each time point. A Dacron polyester-tipped swab was used for vaginal swab sample collection. DPV was extracted from swabs using acetonitrile. After drying under nitrogen, final samples were reconstituted in 500 µL of 60% acetonitrile. Drug content was quantified by LC-MS/MS method as described above.

##### Film Distribution in Genital Tract

In a separate study, three female pigtailed macaques were used to assess distribution of two types of films within the vaginal tract. Bioadhesive films and quick dissolving films were loaded with gadobenate dimeglumine (GD-contrast agent) for MRI tracking of film dispersion at 4 and 24 h post film insertion. MRI scans were conducted on a binary scale signal for film distribution in the vaginal canal, ectocervix, endometrium, fallopian tubes, rectum, urethra, and periurethral tissues. The baseline MRI for each macaque was also recorded before film insertion.

##### Assessment of DPV/LNG Combination Film in Macaques

**Figure 1:**
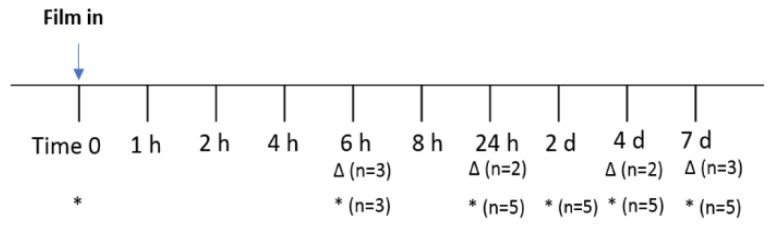
Study design of in vivo assessment of DPV/LNG bioadhesive film for drug delivery. Blood was collected at each time point. (*) indicates the time points for vaginal swab samples collection. (∆) indicates the time points for vaginal and cervical biopsy collection. Films tested: DPV bioadhesive film, LNG bioadhesive film, DPV/LNG combination film.

DPV/LNG single and combination MPT films were studied in three arms. Five female pigtailed macaques were tested in each arm for PK evaluation. The study timeline and biological sample collection points are shown in Figure 1. Briefly, after vaginally administering each film, drug in vaginal fluid was collected by vaginal swab using a Dacron polyester-tipped swab at 6 h, 24 h, 2 days, 4 days, and 7 days after film insertion. The drug extraction method for DPV and/or LNG was the same as described before. Vaginal and cervical biopsies were collected from three macaques at 6 h and collected from the other two macaques at 24 h. Then, at day 4, biopsies were collected from two of the macaques that were biopsied at 6 h, and at day 7 samples were collected from one macaque biopsied at 6 h and two macaques biopsied at 24 h. This collection timeline was chosen to ensure sufficient recovery of macaque cervicovaginal tissue after each biopsy. The extraction solvent for DPV and/or LNG from tissue biopsies was a mixture of methanol, acetonitrile, and methyl *tert*-butyl ether (MTBE). After drying under nitrogen, final samples were reconstituted in 500 µL of 60% acetonitrile. Drug content was quantified by LC-MS/MS method, as described above. Plasma samples were collected at each time point for five macaques. Drug extraction from plasma samples was conducted by protein precipitation, and supernatant was collected for drug analysis by the LC-MS/MS method described above.

#### 2.2.10. Statistical Analysis

All data were presented as either mean ± standard deviation (SD) or median with range. Statistical analyses were conducted using the GraphPad Prism software version 7. Student’s *t*-test was used for testing significance between two groups, with *p* ≤ 0.05 as statistical significance and *p* ≤ 0.001 for high significance.

## 3. Results

### 3.1. Thiolation Degree Determination in Thiomers

Thiolated chitosan was synthesized using chitosan with various molecular weights (Table 2). From thiol degree characterization, a large variability was observed for chitosan with a wide molecular weight range (50–700 kDa). To develop reproducible thiomers from a product development standpoint, chitosan with intermediate molecular weight range (190–310 kDa) was selected. The thiolation was conducted under different pH values of 5, 6, and 7 to assess the effect of pH on the degree of thiolation in chitosan. With an increase in pH, reductions in thiolation degree and solubility were observed. An increased number of thiol groups on thiomers provides increased mucoadhesiveness. Additionally, because film manufacture requires preparation of aqueous solutions of thiomers and other excipients, the aqueous solubility of thiomer is a critical parameter. The results showed that thiomer synthesized at pH 5 displayed the highest thiolation degree and the best water solubility. Thus, thiomer synthesized using medium molecular weight range at pH 5 was chosen for further study.

### 3.2. Mechanism of Polymer and Mucin Interaction

**Figure 2:**
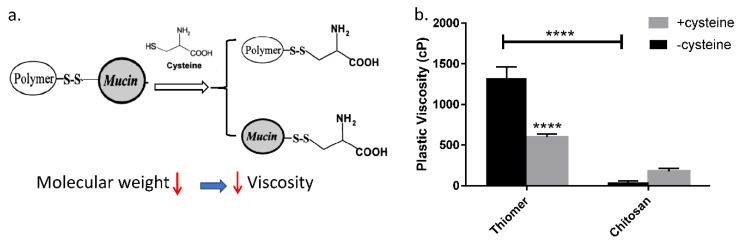
Mechanism of mucoadhesiveness for thiomers. (**a**) Scheme for explanation of the interactions between thiomers and mucins. (**b**) Plastic viscosity evaluation for a mixture of thiomer and mucin with/without adding cysteine and a mixture of chitosan and mucin with/without adding cysteine. Viscosity comparisons were performed between groups of cysteine addition (black bars) and no addition (grey bars) groups. *p* < 0.0001 (****).

The interaction between mucin and thiomers was studied through rheological measurement. As illustrated in Figure 2a, when a polymer interacted with mucin via disulfide bond, the molecular weight of polymer-mucin conjugates increased, which resulted in a solution with higher viscosity. Since L-cysteine competitively interacts with mucin via the same mechanism, the addition of cysteine in the polymer-cysteine conjugates solution broke the conjugates into polymer-cysteine and mucin-cysteine, resulting in solutions with lower viscosity. It was observed that the viscosity of the thiomers and mucin mixture was significantly reduced with the addition of cysteine, as compared to the group without the addition of cysteine. On the contrary, chitosan, a control polymer that has no thiol group, did not show the same trend after adding cysteine to the mixture of chitosan and mucin (Figure 2b). These results indicate that in addition to the electrostatic interactions between mucin and chitosan, thiomer forms disulfide bonds with mucin, which will further strengthen mucoadhesiveness.

### 3.3. Tissue Toxicity of Thiomers

**Figure 3:**
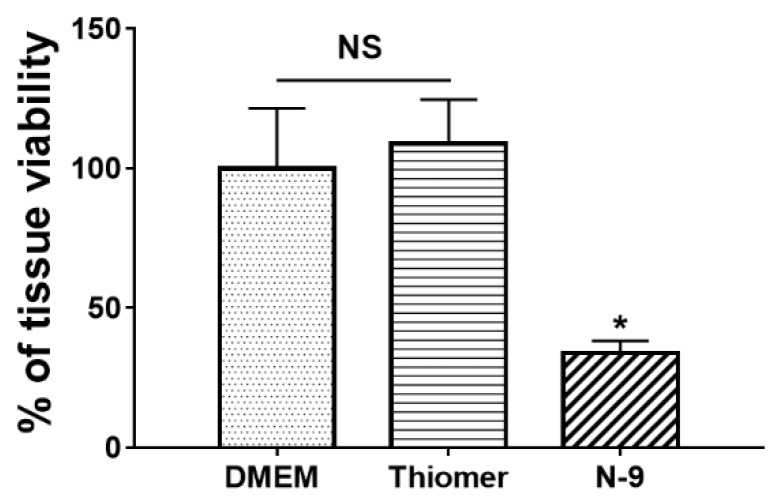
Ex vivo tissue toxicity of thiomers. No significant difference was observed between Dulbecco’s modified Eagle’s medium (DMEM) and thiomers. N-9 was chosen as a positive control. Values indicate mean ± SD with triplicates. NS: No significant difference. (*n* = 3, * *p* < 0.05).

The tissue safety of thiomers was confirmed in human ectocervical tissues using MTT assay. The percentage of tissue viability was determined and showed no significant difference as compared to DMEM, the tissue culture medium (negative control). However, the positive control, N-9, showed significant reduction (*p* < 0.05) in tissue viability 17.18 ± 9.21 after 6 h incubation (Figure 3). Thus, thiomers demonstrated safety in this excised human ectocervical tissue model.

### 3.4. Film Physicochemical Characterizations

DPV quick-dissolving film and DPV/LNG single entity and combination bioadhesive films were manufactured using a solvent casting method. These films were characterized for mass, thickness, water content, drug content, puncture strength, and disintegration, and the results are shown in Table 3. Compared to the quick-dissolving film, all bioadhesive films showed significantly increased puncture strength and disintegration time (*p* < 0.05). In addition, compared to DPV film and combination film, the mass and thickness of LNG film were higher, whereas the water content was lower. The LNG-loaded film had higher puncture strength and disintegration as compared to the DPV film, which might be due to the lower water solubility of LNG. The combination drug-loaded film showed a longer disintegration time than the single drug loaded films.

### 3.5. In Vitro Drug Dissolution

**Figure 4:**
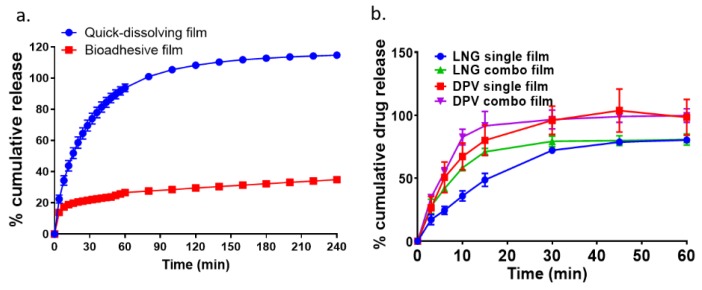
In vitro dissolution of films. (**a**) Cumulative DPV release from quick-dissolving film and bioadhesive film in 1% Cremophor^®^ EL in Milli-Q water. (**b**) Cumulative DPV/LNG release from single entity and combination films in 40% of acetonitrile in Milli-Q water.

The release kinetics of DPV bioadhesive film was compared to the quick-dissolving film, as shown in Figure 4a. In total, 100% of DPV was released within 60 min for quick-dissolving film, whereas the bioadhesive film only showed 27.61% ± 0.26% DPV release in the same time frame, which indicated that thiomer incorporation resulted in a significantly slower drug release rate as compared to film without thiomer modification. To compare the drug dissolution profile for single and combination films, a modified in vitro USP IV apparatus dissolution method using 40% of acetonitrile as the dissolution medium was applied. As shown in Figure 4b, from the same film, DPV released faster than LNG, either for single or combination drug loaded film (*p* < 0.05). There was no significant difference in the extent of release for DPV and LNG between their single and combination films, respectively. The method applied to determine drug release was developed as a product quality control test and does not predict release in the biological compartment.

### 3.6. Ex Vivo Tissue Mucoadhesiveness

**Figure 5:**
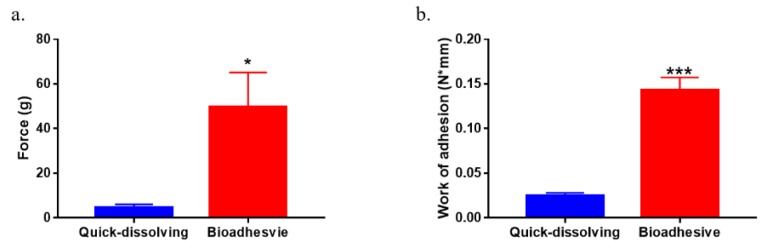
Ex vivo mucoadhesiveness of quick-dissolving and bioadhesive films on porcine intestinal mucosal tissues. (**a**) Tensile force for detachment of quick-dissolving and bioadhesive films from mucosal tissues. (**b**) Work of adhesion (area under the curve of tensile force vs. distance) as measurement of mucoadhesiveness of quick-dissolving and bioadhesive films (*n* = 4, * *p* < 0.5, *** *p* < 0.005).

Film tissue mucoadhesion was evaluated by a texture analyzer using porcine intestinal tissue. The results of the tensile force and work of adhesion for quick-dissolving film and bioadhesive film are shown in Figure 5a,b, respectively. The peak force for thiomer film detaching from mucosal tissue was 9.7-fold higher than the quick-dissolving film (*p* < 0.05). Also, the work of adhesion increased 4.8-fold after incorporation of thiomers in the film matrix (*p* < 0.005).

### 3.7. In Vivo Retention and Safety of Bioadhesive Films in Macaque Genital Tract

**Figure 6:**
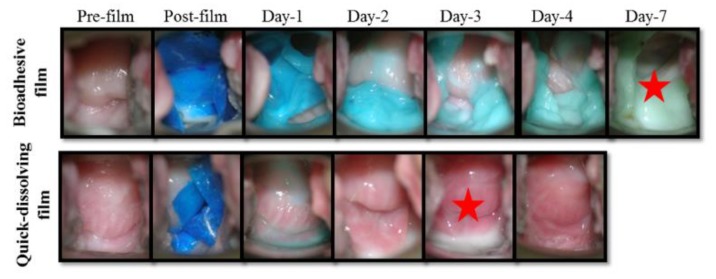
Tissue retention and distribution of bioadhesive film and quick-dissolving film in macaque model. Representative colposcope image for blue dye loaded bioadhesive film and quick-dissolving film in macaque vaginal compartment (red star marks the last presence of film in vaginal cavity).

In vivo retention of the quick-dissolving and bioadhesive films was compared by colposcopic visualization. Colposcopy images were taken at days 1, 2, 3, 4, and 7 after the film insertion. Representative colposcopy images shown in Figure 6 demonstrate that the bioadhesive film was retained in the vaginal compartment for up to 7 days, whereas the quick-dissolving film disappeared at day 4. No visual changes in tissue integrity for each colposocpic check were observed in the vagina after the film insertion, suggesting that the bioadhesive film was safe. In addition, vaginal swabs were taken to evaluate the in vivo drug release from the bioadhesive and quick-dissolving films into vaginal fluid. DPV was detectable in the vaginal fluid for the bioadhesive film at day 7, whereas no DPV was detected for the quick dissolving film at day 7 (Table 4).

### 3.8. Distribution of Bioadhesive Films in Macaque Genital Tract

**Figure 7:**
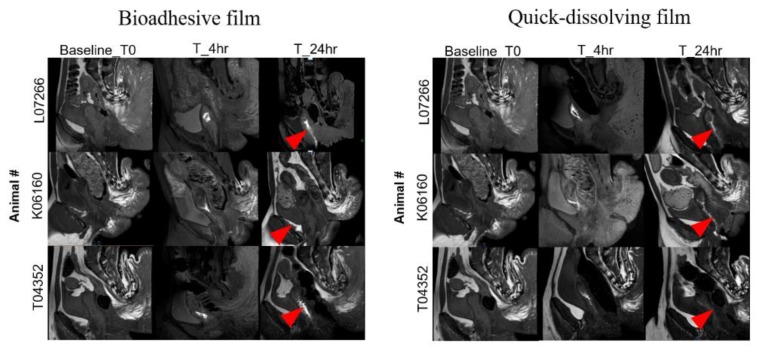
Distribution of bioadhesive and quick-dissolving films in macaque vaginal cavity (red arrow indicates the dispersion of film, *n* = 3).

The distribution of gadolinium-containing bioadhesive films was evaluated by MRI in the macaque genital tract at 4 and 24 h post-insertion (Figure 7). At 4 h post film insertion, the bioadhesive film showed a more localized distribution at the site of insertion in the vaginal cavity, whereas the quick-dissolving film showed a diffuse pattern indicating film distribution through most of the vaginal lumen. At 24 h, the bioadhesive film was distributed throughout the vaginal lumen with intense contrast. This result was expected due to the super-disintegration nature of the quick-dissolving film compared to the bioadhesive film, which is consistent with the data shown in Table 3 and Figure 5a. No contrast was noted in the genital tract other than the vaginal compartment at any time point (Figure 7).

### 3.9. Local and Systemic PK of DPV/LNG Bioadhesive Film

**Figure 8:**
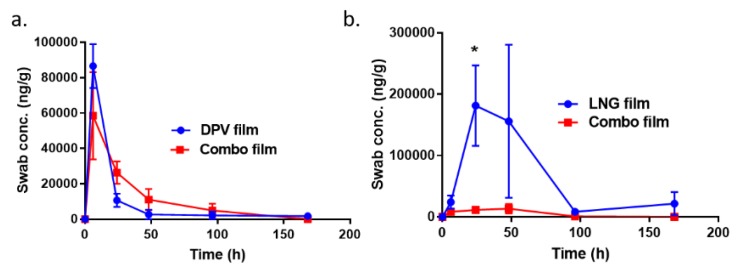
In vivo assessment of DPV/LNG single entity film and combination film in pigtailed macaque models. (**a**) DPV drug level in vaginal swab over time for single film and combination film. (**b**) LNG drug level in vaginal swab over time for single film and combination film (* *p* < 0.5).

After proving that the bioadhesive film was able to be retained on the vaginal mucosa for up to 7 days in vivo, DPV and LNG were loaded into the bioadhesive film for evaluation of local and systemic PK profile. Vaginal swabs were utilized to sample the drug released in the vaginal fluid for DPV/LNG single and combination films. The amount of the drug collected onto the vaginal swab was determined using LC-MS/MS (Figure 8a,b). There was no significant difference for the DPV release profile into the vaginal fluid between DPV single and DPV/LNG combination films. The maximum DPV level was obtained at 6 h after film insertion for both films. The DPV concentration in vaginal fluid at day 7 for both films was detectable (lower limit of quantification (LLOQ) = 0.2 ng/mL) and at least threefold higher than its cellular IC_50_ (7.9 ng/mL) (Figure 8a). LNG showed significantly higher concentration in vaginal fluid for the LNG single entity film than that observed for the DPV/LNG combination film (*p* < 0.5). The maximum concentration at 24 or 48 h for both single entity and combination LNG films was obtained after vaginal administration. However, LNG release from either the single entity or combination LNG films was not time-dependent (Figure 8b). The LNG single film showed detectable LNG levels (LLOQ = 0.2 ng/mL) at day 7 and the DPV/LNG combination film showed detectable LNG levels at day 4, with one macaque showing detectable LNG levels at day 7. It was also observed that a greater amount of LNG was detectable in the vaginal fluid than DPV for single films, which indicated that the vaginal mucosal clearance of DPV could be faster than that of LNG.

The vaginal and cervical tissue drug levels were also measured, and the results are listed in Table 5 and Table 6. It was observed that the highest DPV concentration in the vaginal tissue was observed at 6 h for DPV single entity and DPV/LNG combination films. The maximum DPV level in cervical tissue was found at 6 h for the single entity DPV film. The combination film showed comparable cervical tissue DPV level at 6 h, 24 h, and day 4, and reduced level at day 7. At day 7, DPV levels for single and combination films were 1–2 log higher than their IC_50_ for both vaginal and cervical tissues. The highest LNG concentration in vaginal tissues was observed at 24 h for LNG single film and at 6 h for DPV/LNG combination film. The LNG single entity film showed comparable LNG level in cervical tissue at 6 h, 24 h, and day 4, and decreased at day 7. Both drugs were detectable at day 7 in vaginal and cervical tissues, either for single or combination films, which indicated that the bioadhesive film was able to deliver DPV or LNG individually or simultaneously to tissues for at least 7 days.

**Figure 9:**
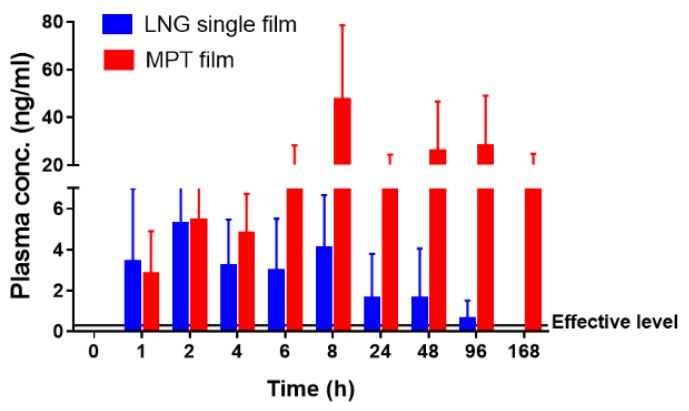
Plasma levels of LNG for single and combination bioadhesive films. Data were analyzed using paired *t*-tests for each time point. (* *p* < 0.05, *n* = 5).

In addition to the local PK for DPV and LNG, drug plasma levels were also assessed. Minimum systemic exposure of DPV was observed for DPV single and DPV/LNG-combination films. LNG plasma levels were observed within 1–2 h post film dosing and achieved 1 log higher than the minimum LNG plasma concentration required for effective contraception (0.3 ng/mL) [43]. The peak plasma concentration for LNG was obtained at 8 h for both films (combination: 2 logs higher than 0.3 ng/mL). As compared to the single LNG film, LNG MPT film showed significantly (*p* < 0.05) higher plasma exposure at all the time points (Figure 9). Furthermore, when co-delivering with DPV, the plasma level of LNG persisted for up to 7 days.

## 4. Discussion

MPT products offer the advantage of simultaneously meeting multiple sexual and reproductive health needs, such as contraception and protection against sexually transmitted infections (STIs) including HIV [44]. The polymeric film is a well-accepted dosage form that has been developed for delivering combinations of antiretroviral drugs [19,45]. Clinically advanced vaginal films have been developed for quick drug release as coitally-dependent products. Dosage forms that require less frequent administration are preferred by many users and as such are promising products for enhancing use compliance among these users [46]. The primary objective of this study was to evaluate the feasibility of MPT bioadhesive films for co-delivering an antiretroviral drug (DPV) and progestin (LNG) as a novel, weekly-administered MPT product.

The polyvinyl alcohol-based quick-dissolving film has been previously evaluated in clinical studies. In these studies it was shown to have a favorable acceptability and safety profile. The feasibility of formulating DPV in this film platform was also confirmed in these studies. Thus, it was reasonable to utilize this film as a platform to explore more advanced applications such as bioadhesion. The DPV-containing bioadhesive film was assessed for physicochemical properties including appearance, weight, and thickness, as well as water content, drug content, puncture strength, and disintegration. Compared to the quick-dissolving film, the incorporation of thiomer significantly increased the puncture strength and disintegration time of the film. The thiolated chitosan synthesized in this study had high molecular weight and low water solubility. Thus, the incorporation of thiolated chitosan could decrease the overall water solubility of the film. In addition to the DPV film, LNG and the combination of LNG/DPV were also formulated in the bioadhesive films. Within the physicochemical assessment, it was found that there was no significant difference between the single entity film and combination film. This was because both DPV and LNG are hydrophobic small molecules, which are unlikely to have significant impact on the film matrix.

Thiomer has been reported as an advanced bioadhesive polymer due to its covalent bond formation with mucin via thiol groups [25]. This property was confirmed when it was incorporated in our polymeric film matrix using an ex vivo quantitative tensile method. The mucoadhesion of the bioadhesive film increased approximately fivefold after incorporating thiomers. The in vivo film retention in vaginal tissue was also evaluated in a pigtailed macaque model using colposcopy imaging and MRI. This is the first time the in vivo residence time of thiomer-containing formulations was investigated in the vaginal compartment. Two out of five pigtailed macaques showed that the bioadhesive film was retained in the vaginal compartment for at least 7 days. The complete loss of the film for the other three macaques at day 1 or day 3 might have been due to self-grooming, which has been previously observed in some macaques. Thiomer is also a permeation enhancer, as reported in multiple formulations [47,48], which necessitates evaluating its distribution in different parts of the female genital tract. However, our MRI results conclusively showed no evidence of contrast for bioadhesive films in the ovaries, upper uterus, or rectum. This suggests that the distribution of bioadhesive film was confined to the vaginal lumen.

In addition to the film persistence observed in vivo, the DPV release from the bioadhesive film was sustained in vivo as compared to the quick-dissolving film. The in vivo release pattern (Table 4) for DPV from the bioadhesive and quick-dissolving film was consistent with the in vitro data (Figure 4). The sustained drug release rate can be attributed to the addition of thiomers, which increased the disintegration time of the film significantly (Table 3). The observed longer disintegration time can be explained by the high content of cross-linked disulfide bonds within the polymeric network [49]. The increased disintegration time indicated that water took more time to penetrate into the bioadhesive film matrix, which slowed the erosion process, thereby achieving a lower release rate [50]. In the in vivo study, a 7 day release profile for the DPV bioadhesive film was observed, which demonstrated the potential of this film platform to be developed into a weekly MPT product.

After confirming that this film platform can be used to deliver drug for 1 week, DPV and LNG were co-formulated into the bioadhesive film as a potential MPT product and further evaluated in vivo. The investigation of local and systemic PK of the antiretroviral drug and progestin is regarded as a very important step in the development of MPT product [51]. The mechanism of action for DPV demands sufficient concentration in the target mucosal tissue, which is the major site for sexually transmitted HIV infection. A study of the LNG intrauterine device not only showed the systemic effectiveness in inhibition of ovulation via binding to the progesterone and estrogen receptors, but also demonstrated local effectiveness via cervical mucus thickening, which inhibits sperm motility [35]. Thus, for intravaginal delivery of LNG, tissue concentration and systematic circulation are both desirable.

Our data showed sufficient DPV concentration in vaginal fluid, and vaginal and cervical tissues even with co-delivery of LNG. Minimal systemic exposure of DPV was detected at each time point. Slightly higher, but not statistically significant, tissue DPV level was observed for the combination film as compared to the DPV single entity film. This increase may have been due to the lower vaginal mucosal clearance of DPV for the DPV/LNG combination film. A similar result was also reported by Akil A. et al. where the co-delivery of DPV with tenofovir increased the tissue accumulation of DPV in the ex vivo human ectocervical tissue explant model [45]. These results indicate that co-delivery could have an impact on the local PK of drug. In our study, DPV and LNG showed different PK profiles with co-delivery. LNG displayed lower drug concentration in the vaginal fluid for the combination film as compared to the single entity film, which could result in slightly lower vaginal and cervical tissue concentration observed for the combination film (not statistically significant due to small sample number). However, LNG combination film showed significantly higher drug concentration in the plasma compared to the single entity film. These results indicate that drug–drug interaction may also occur during the co-delivery of anti-retroviral drugs and contraceptives.

One of the possible explanations for the drug–drug interaction during the co-delivery of DPV and LNG is the involvement of cytochrome P-450 (CYP450) enzymes. Multiple studies have shown that CYP3A4 is expressed in vaginal and cervical tissues [52,53,54]. LNG is a substrate of cytochrome P-450 isoenzyme 3A4 (CYP3A4) [55], and DPV can stimulate the increase of CYP3A4 expression at mRNA levels [52]. Thus, when co-delivered with DPV, the metabolism of LNG could be increased in the vaginal and cervical tissues. This could result in the decreased vaginal and cervical tissue levels of LNG that were observed in our study. On the other hand, LNG is an inhibitor of CYP2C19 and CYP3A4 [36], whereas DPV is the substrate of both enzymes that are also expressed in vaginal tissues. Although LNG’s inhibitory activity for these enzymes is weaker than other progestins [56], the inhibition of CYP3A4 and CYP2C19 could increase DPV levels in vaginal and cervical tissues. Moreover, both DPV and LNG have high protein binding properties, which could play a role in the variable drug exposure observed with combination MPT films. The increased plasma drug level, especially for LNG after co-delivery, could be due to the competitive protein binding; however, this needs to be further studied. Our results indicate potential drug–drug interactions, which require further exploration given that DPV and LNG combination products are also being pursued in a vaginal ring platform.

## 5. Conclusions

In conclusion, this study reported a novel MPT bioadhesive film platform for delivering antiretroviral drug and progestin simultaneously, and evaluated the PK profile of the co-delivery of DPV and LNG in a non-human primate model. In this study, 1 week retention in the vaginal compartment was demonstrated in vivo for the designed thiomer-containing films. This study also demonstrated the ability of film platform to deliver LNG intravaginally with or without combination of DPV. The co-delivery of DPV and LNG showed different PK profiles as compared to single entity films, which indicated potential drug–drug interactions occurring locally or systemically. These results support further investigation of a DPV and LNG-containing film as an MPT product for combined prevention of HIV and unintended pregnancy. In order to advance this platform, future studies to investigate long-term stability of the developed MPT platform are warranted. Although numerous reports on thiomers have been published, they have not been extensively evaluated in the vaginal compartment, and as such lack toxicity data. Therefore, the effect of this polymer on innate protective factors such as microbiome and glycome requires further investigation.

## Figures and Tables

**Table 1:** Film formulation of quick-dissolving film and bioadhesive film. DPV: dapivirine; LNG: levonorgestrel.

Component	Bioadhesive Film (*w*/*w* %)	Quick-Dissolving Film (*w*/*w* %)
Milli-Q water	86.10	83.6
Polyvinyl alcohol 40-88	7.02	7.02
Polyethylene glycol 8000	2.34	2.34
Methocel E5	1.75	1.75
Sodium starch glycolate	0	3.5
Thiomer	1	0
Glycerin	0.73	0.73
Propylene glycol	0.73	0.73
Drug (DPV/LNG)	0.34	0.34
Total	100.00	100.00

**Table 2:** Characterization of thiomers.

Chitosan (MWR, kDa)	Synthesis pH	Thiol Degree (µmol/g)	Rate of Solubility in Water
50–700	5	183.45 ± 130.29	+++
190–310	5	182.94 ± 31.44	+++
190–310	6	61.83 ± 13.31	+
190–310	7	71.26 ± 27.11	+

MWR: molecular weight range; +++: dissolves in water very fast (less than 1 h); +: dissolves in water slow (more than 8 h).

**Table 3:** Physicochemical characteristics of quick-dissolving film and bioadhesive multipurpose prevention technologies (MPT) films.

Characterizations	DPV Quick-Dissolving Film	DPV Bioadhesive Film	LNG Bioadhesive Film	DPV/LNG MPT Bioadhesive Film
Appearance	White, transparent, smooth, and soft	White, transparent, smooth, and soft	White, transparent, smooth, and soft	White, transparent, smooth, and soft
Weight (mg)	69.20 ± 1.76	69.35 ± 5.9	79.14 ± 8.8	65.85 ± 3.93
Thickness (µm)	155.90 ± 9.75	110.75 ± 12.06	127.5 ± 11.8	105.28 ± 6.96
Water Content % (*w*/*w*)	4.87 ± 0.20	6.68 ± 0.43	4.95 ± 0.17	6.36 ± 0.50
Drug content (mg/film)	1.41 ± 0.13	1.68 ± 0.15	1.80 ± 0.09	1.71 ± 0.15/DPV 1.50 ± 0.13/LNG
Drug loading % (*w*/*w*)	2.04 ± 0.19	2.42 ± 0.22	2.27 ± 0.11	2.60 ± 0.23/DPV 2.28 ± 0.20/LNG
Puncture Strength (Kg/mm)	3.77 ± 0.58	12.41 ± 0.44	14.18 ± 2.28	13.03 ± 0.50
Disintegration (sec)	56.36 ± 6.49	153.12 ± 33.81	211.33 ± 70.52	228.44 ± 77.46

**Table 4:** In vivo release of DPV from bioadhesive film and quick-dissolving film formulations.

	Bioadhesive Film			Quick-Dissolving Film		
Days of Detection	Number of Macaques	% above LLOQ	Median (Range)	Number of Macaques	% above LLOQ	Median (Range)
Day 1	5	100	12.3 (2.87–36.71)	5	100	18.56 (10.18–69.92)
Day 2	5	100	2.62 (0.91–24.63)	5	100	1.68 (1.51–2.85)
Day 3	5	100	0.49 (0.21–11.32)	5	100	0.49 (0.4–1.62)
Day 4	5	100	0.40 (0.11–20.03)	5	100	0.21 (0.11–0.52)
Day 7	5	40	33.01 (0.1–64.41)	ND	ND	ND

ND: not detectable; LLOQ: lower limit of quantification.

**Table 5:** DPV concentration in vaginal swab, and vaginal and cervical tissue for DPV single and DPV/LNG combo films.

DPV	6 h			Day 1			Day 4			Day 7		
	Number of Macaques	% above LLOQ	Median (Range)	Number of Macaques	% above LLOQ	Median (Range)	Number of Macaques	% above LLOQ	Median (Range)	Number of Macaques	% above LLOQ	Median (Range)
Vaginal swab												
Single film	3	100	86744.53 (64936.83–108126.9)	5	100	11327.68 (664.89–19245.81)	5	80	82.80 (0.1–8656.03)	5	60	1.53 (0.1–5339.14)
Combo film	3	100	49154.43 (21220.13–105228.6)	5	100	27022.57 (4573.604–41360.52)	5	100	2039.18 (81.76–20154.96)	5	80	26.34 (0.1–34.133)
Vaginal tissue												
Single film	3	100	6425 (2571–13071)	2	100	925.5 (107–1744)	2	100	1768 (361–3175)	3	100	215 (101–735)
Combo film	3	66.7	21238 (0.1–213096)	2	100	10331.5 (7452–13211)	2	100	2851.5 (1759–3944)	3	66.7	495 (0.1–605)
Cervical tissue												
Single film	3	100	2424 (359–4138)	2	100	297.5 (264–331)	2	100	478 (141–815)	3	100	342 (201–626)
Combo film	3	100	5390 (2959–10194)	2	100	6698 (2206–11190)	2	100	5581 (3258–7904)	3	100	93.9 (32.7–1943)

Single film: DPV-containing film; combo film: DPV and LNG-containing film; values are presented as ng/g for vaginal swab, ng/g for vaginal and cervical tissue.

**Table 6:** LNG concentration in vaginal swab, and vaginal and cervical tissue for LNG single and DPV/LNG combo films.

LNG	6 h			Day 1			Day 4			Day 7		
	Number of Macaques	% above LLOQ	Median (Range)	Number of Macaques	% above LLOQ	Median (Range)	Number of Macaques	% above LLOQ	Median (Range)	Number of Macaques	% above LLOQ	Median (Range)
Vaginal swab												
Single film	3	100	16819.47 (10601.74–45273.93)	5	100	12623.8 (43223.21–411886.5)	5	100	2888.28 (50.26–29221.25)	5	100	4663.56 (84.98–97194.82)
Combo film	3	100	6533.28 (4390.29–14106.52)	5	100	11317.51 (210.43–16382.36)	5	100	12.52 (3.11–3703.99)	5	20	0.1 (0.1–2.81)
Vaginal tissue												
Single film	3	100	1631 (444–19086)	2	100	21344 (13300–29388)	2	100	3901.5 (1062–6741)	3	100	188 (103–226)
Combo film	3	100	5548 (473–132699)	2	100	1785 (1168–2402)	2	100	303 (102–504)	3	66.7	264 (0.1–679)
Cervical tissue												
Single film	3	100	7258.5 (3059–11458)	2	100	7582.5 (6759–8406)	2	100	9335.5 (4620–14051)	3	100	154 (118–166)
Combo film	3	100	2771.85 (2273–3450)	2	100	1987.5 (873–3102)	2	100	901.42 (554–1248.84)	3	66.7	13.9 (0.1–222)

Single film: LNG-containing film; combo film: DPV and LNG-containing film; values are presented as ng/g for vaginal swab, ng/g for vaginal and cervical tissue.
